# Long-term inhaled corticosteroid treatment and severe asthma in children - the impact on body height and weight

**DOI:** 10.1186/2045-7022-5-S2-P15

**Published:** 2015-03-23

**Authors:** Guergana Petrova, Vera Papochieva, Snezhina Lazova, Dimitrinka Miteva, Ljupco Zafirovski, Lihnida Zafirovska-Matovska, Penka Perenovska

**Affiliations:** 1 UH "Alexandrovska", Pediatric clinic, Sofia, Bulgaria; 2 Children’s Hospital for Respiratory Diseases and TB, Respiratory departement, Skopje, Macedonia; 3 Sana-Klinik, Pediatric department, Bergen, Germany

## Background

Chronic conditions such as asthma are usually stigmatized as one of the reasons for short stature. A widespread belief also is that prolonged long-term treatment with corticosteroids will lead to elevated body weight and also could lead to short stature.

Therefore we evaluated the height and weight status in the children admitted in the hospital for asthma attack regarding the asthma severity, the natural history of asthma and inhaled corticosteroid (ICS) treatment period.

## Material and method

For a period of 6 months (Jan- Jun 2014) we evaluated 100 (54 boys, mean age 7,48±0.53 and 46 girls, mean age 7.66±0.64) children admitted for asthma attack. A detailed natural history, disease summary, pulmonary function test, bronchodilation test and anthropometric data were gathered, as well as data for a control group of 132 patients with acute respiratory infections matched by age and sex.

## Results

49 asthmatic patients have been using ICS (18 for more than 5 years). There were not any significant correlations between height, weight, BMI (SDS) and the ICS treatment period, the time without controller therapy, BDR response, birth weight, the asthma duration, family history or atopy status (p>0,05, r 0,01-0,2). No differences were found in height SDS when the kids were stratified according ICS treatment and its duration (less or over 5 years)(p>0,1), the same results came for weight (p>0,1) and BMI (p>0,1). Comparing with the control group, there is not a significant difference in weight (p=0,07 asthma vs controls, p=0,15 asthma on ICS vs controls), height (p=0.06 asthma on ICS vs. controls). Severe asthma patients (11 on high dose combined corticosteroids ± montelucast) also don't show any significant difference in height, BMI and weight compared to other asthma patients (p>0,5). Long-term not treated asthmatics also don't have any significant difference in anthropometric data compared to other asthmatics.

## Conclusion

Long-term ICS therapy or not proper treatment is not that determining for height and weight status in asthma patients. Interesting future study would determine whether there is a correlation between different food behavior and exercise pattern that could lead the previously published data.

